# Exploring Social Media Posts on Lifestyle Behaviors: Sentiment and Content Analysis

**DOI:** 10.2196/65835

**Published:** 2025-06-25

**Authors:** Yan Yee Yip, Mohd Ridzwan Yaakub, Mohd Makmor-Bakry, Muhammad Iqbal Abu Latiffi, Wei Wen Chong

**Affiliations:** 1 Centre for Quality Management of Medicines, Faculty of Pharmacy, Universiti Kebangsaan Malaysia, Jalan Raja Muda Abdul Aziz, Kuala Lumpur, 50300, Malaysia, 60 392897265; 2 Centre for Clinical Epidemiology, Institute for Clinical Research, National Institutes of Health, Ministry of Health Malaysia, Setia Alam, Shah Alam, Malaysia; 3 Center for Artificial Intelligence Technology, Faculty of Information Science & Technology, Universiti Kebangsaan Malaysia, Bangi, Selangor Darul Ehsan, Malaysia

**Keywords:** consumer health information, content analysis, chronic illness, health promotion, healthy lifestyle, lifestyle, lifestyle risk reduction, internet, primary prevention, sentiment analysis, social media

## Abstract

**Background:**

There has been an increase in the prevalence of noncommunicable diseases in Malaysia. This can be prevented and managed through the adoption of healthy lifestyle behaviors, including not smoking, avoiding alcohol consumption, maintaining a balanced diet, and being physically active. The growing importance of using social media to deliver information on healthy behaviors has led health care professionals (HCPs) to lead these efforts. To ensure effective delivery of information on healthy lifestyle behaviors, HCPs should begin by understanding users’ current opinions about these behaviors and whether the users are receptive to recommended health practices. Nevertheless, there has been limited research conducted in Malaysia that aims to identify the sentiments and content of posts, as well as how well users’ perceptions align with recommended health practices.

**Objective:**

This study aims to examine social media posts related to various lifestyle behaviors, by using a combination of sentiment analysis to analyze users’ sentiments and manual content analysis to explore the content of the posts and how well users’ perceptions align with recommended health practices.

**Methods:**

Using keywords based on lifestyle behaviors, posts originating from X (formerly known as Twitter) and published in Malaysia between November and December 2022 were scraped for sentiment analysis. Posts with positive and negative sentiments were randomly selected for content analysis. A codebook was developed to code the selected posts according to content and alignment of users’ perceptions with recommended health practices.

**Results:**

A total of 3320 posts were selected for sentiment analysis. Significant associations were observed between sentiment class and lifestyle behaviors (*χ*^2^_6_=67.64; *P*<.001), with positive sentiments higher than negative sentiments for all lifestyle behaviors. Findings from content analysis of 1328 posts revealed that most of the posts were about users’ narratives (492/1328), general statements (203/1328), and planned actions toward the conduct of their behavior (196/1328). More than half of tobacco-, diet-, and activity-related posts were aligned with recommended health practices, whereas most of the alcohol-related posts were not aligned with recommended health practices (63/112).

**Conclusions:**

As most of the alcohol-related posts did not align with recommended health practices, the findings reflect a need for HCPs to increase their delivery of health information on alcohol consumption. It is also important to ensure the ongoing health promotion of the other 3 lifestyle behaviors on social media, while continuing to monitor the discussions made by social media users.

## Introduction

Noncommunicable diseases (NCDs) have become a significant global health challenge, accounting for 74% of all deaths worldwide [1]. NCDs have also become a growing public health concern in middle-income countries within the Southeast Asia region. Malaysia is one of the countries in the region that has been significantly impacted by NCDs, with over 67% premature NCD-related mortality and over 70% disease burden [2]. Approximately, 2.5% of Malaysian adults, which accounts for over half a million people were affected by all 4 major NCDs, which are diabetes, hypertension, hypercholesterolemia, and obesity [3].

The World Health Organization (WHO) has identified 4 key modifiable risk factors associated with an elevated risk of NCDs, which are tobacco smoking, harmful use of alcohol, unhealthy diet, and physical inactivity [1,4]. The adoption of healthy lifestyle behaviors, including not smoking, avoiding alcohol consumption, maintaining a balanced diet, and being physically active can reduce modifiable risk factors, effectively preventing and managing NCDs. However, national surveys have indicated that the actual adoption of healthy behaviors among Malaysians remains low. For example, over 84% of Malaysian adults were inactive in sports, with half of the population leading a sedentary lifestyle, spending more than 2 hours sitting while awake [3].

In this regard, it is important to deliver information on healthy lifestyle behaviors, with health care professionals (HCPs) being ideally positioned to lead these efforts. Various technologies, such as mHealth applications, wearable devices [5], and social media platforms, can support the delivery of health information. Social media platforms have been widely used for health information delivery as these platforms are accessible to larger populations at a lower cost [6,7]. To effectively promote healthy lifestyle behaviors on social media, HCPs could begin by understanding users’ current opinions on lifestyle behaviors and whether they are receptive to recommended health practices [8]. This could be achieved by examining social media posts discussing on lifestyle behaviors. X (formerly known as Twitter) is one of the microblog-based social media platform that allows users to freely express their opinions through posts, previously referred to as tweets. As of January 2024, approximately 5.71 million social media users in Malaysia were on X, accounting for 16.5% of the country’s population [9], which highlights the growing popularity of X among Malaysians.

When users express their opinions in writing on social media, a range of emotions may be conveyed. Sentiment analysis is the process of classifying this textual data based on the emotions conveyed within the text as positive, negative, or neutral sentiments [10]. It can be conducted through manual annotation of posts or computational approaches [11]. Computational approaches in sentiment analysis are preferred as they are more cost-efficient, and can leverage large amounts of publicly accessible and concise real-time data across different regions and demographics [12]. Methodologies of computational approaches include lexicon-based sentiment analysis, which uses pre-existing dictionaries containing words with pre-assigned sentiment scores of positive, negative, or neutral. Lexicon-based sentiment analysis is effective when limited labelled training data is available with a strong association of sentiments with specific words. The usage of this approach has been documented in numerous studies that analyze sentiments regarding lifestyle behaviors such as the examination of policies on electronic cigarettes [13], vegan-related posts [14], and organic food posts [15].

Lexicon-based sentiment analysis, however, may have limited coverage in terms of vocabulary and often misses sarcasm or irony [16]. Positive sentiments may not necessarily translate to good health practices and vice versa. For example, the sentence “I love tobacco” showed positive sentiments, but the actual context is related to the user’s preference towards unhealthy lifestyle behaviors. In order to further understand the topics communicated on social media and how users’ perceptions are aligned with recommended health practices, lexicon-based sentiment analysis can be supported with manual content analysis. A codebook can be used to manually assign labels to each post, which will provide a more in-depth analysis of the posts [17].

The examination of social media posts across multiple lifestyle behaviors of tobacco smoking, alcohol consumption, diet, and physical activity could facilitate effective comparisons of findings across these different behaviors. The use of such findings would enable HCPs to use social media to deliver information on healthy behaviors by targeting areas where the lifestyle behaviors are not aligned with recommended health practices. Analyses that are focused within a geographic location would provide opportunities for HCPs to prioritize region-targeted health information delivery on social media. Such health information could also potentially be replicated in other countries with similar digital and health ecosystems.

Nevertheless, there have been limited studies that used the combined approaches of lexicon-based sentiment and content analysis to examine social media posts on lifestyle behaviors. The majority of the available studies were focused on other health-related issues such as the examination of users’ perceptions on diabetes [18] and marijuana usage [19]. A study by Kasson et al [20] have used both sentiment analysis and manual content analysis to examine users’ vaping behaviors. However, the study was confined to vaping behaviors during the e-cigarette or vaping use-associated lung injury outbreak and did not address other lifestyle behaviors. In addition, there is a lack of studies examining the opinions of social media users in Malaysia on lifestyle behaviors, despite the increasing burden of NCDs and the rising prevalence of unhealthy lifestyle behaviors in the country.

Therefore, this study used a combination of lexicon-based sentiment analysis and manual content analysis to understand the discussions on lifestyle behaviors among social media users in Malaysia. This study had three objectives: (1) to determine the sentiments of social media users in Malaysia regarding lifestyle behaviors, (2) to identify the content of posts and ascertain if users’ perceptions were aligned with recommended health practices, and (3) to explore the associations between the alignment of users’ perceptions with recommended health practices and sentiment class.

## Methods

### Overview

**Figure 1.**
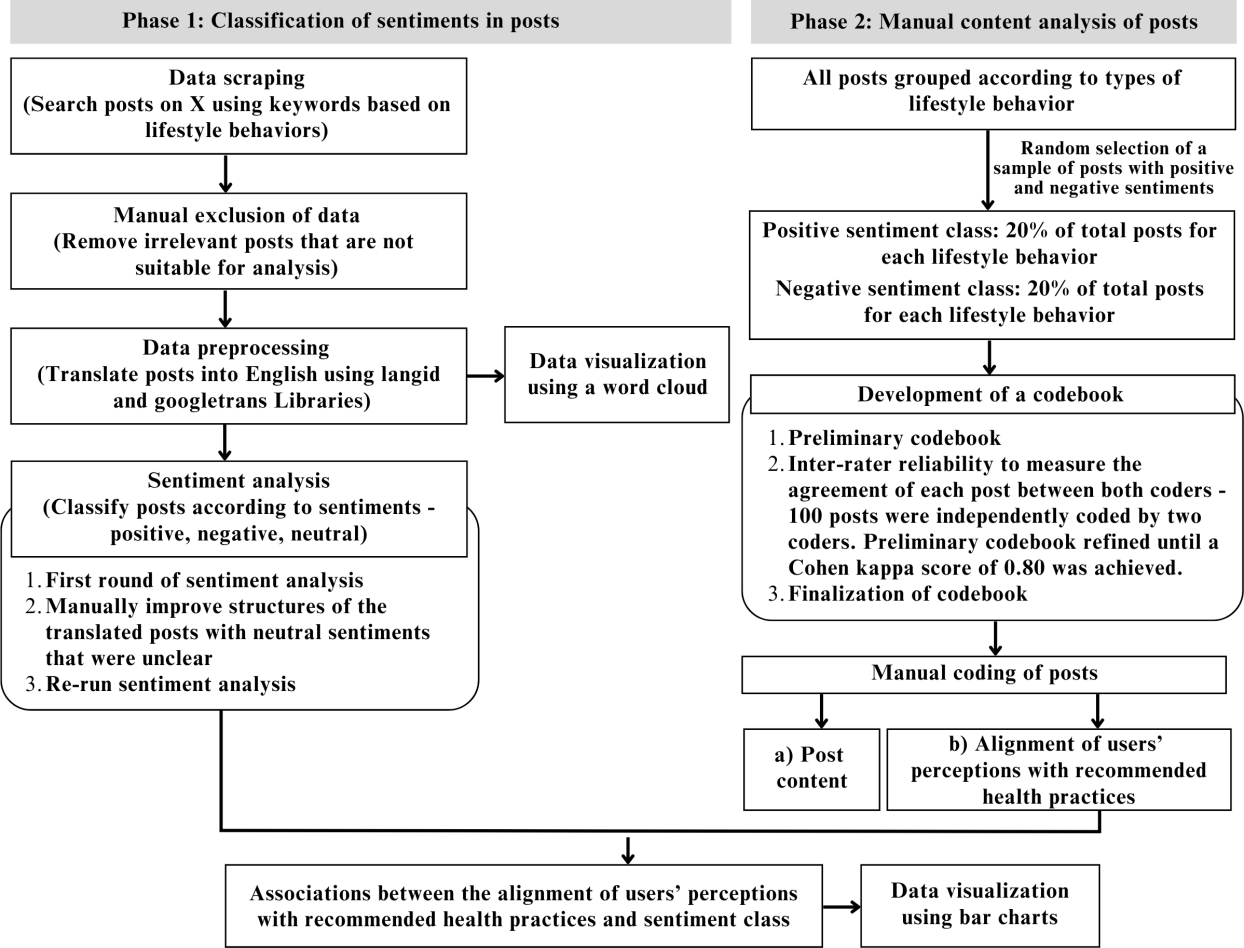
Overall study methods.

Figure 1 shows the overall study methods. In the classification of sentiments in posts, data was scraped from X. Following the manual exclusion of irrelevant posts, the data was cleaned, preprocessed and analyzed for sentiments. Data visualization was subsequently conducted. In the manual content analysis of posts, a random selection of posts with positive and negative sentiments for each lifestyle behavior was manually coded to identify the content of posts and the alignment of users’ perceptions with recommended health practices. Associations between the alignment of users’ perceptions and sentiment class were subsequently explored.

### Classification of Sentiments in Posts

#### Data Scraping

The automated process of extracting large amounts of data from X is known as data scraping. All posts with keywords related to the 4 lifestyle behaviors aimed at reducing the 4 key modifiable risk factors for NCDs were scraped. These keywords are related to tobacco and its derivative products, alcohol, dietary, and physical activity. The 4 sets of keywords are provided in Multimedia Appendix 1. The keywords were derived from published systematic reviews on the management of lifestyle behaviors using social media [21,22]. Additional keywords commonly used locally were added upon discussion with all researchers.

Posts spanning 2 consecutive months were selected. This time frame was deemed to be appropriate, as similar studies analyzing health-related sentiments on the X platform have also utilized data across a 2-month period [23,24]. The selection of the 2 consecutive months was conducted by initially scraping all posts from January to December 2022 according to each month. The 2 months with the highest number of posts were from November to December 2022. Post based in Malaysia were determined using longitude and latitude metadata (4.2105°N, 101.9758°E). In terms of language, posts in Malay and English were scraped. Malay is the national language of Malaysia, whereas English is widely spoken and understood by Malaysians. They also use a mix of both languages, resulting in multilingual data.

Data scraping was conducted separately for each set of keywords using the SNScrape library on Python. In addition to posts, other X metadata such as timestamp, X username, number of reposts, language, and location were also collected.

#### Manual Exclusion of Data

All posts were manually screened by 2 researchers to exclude those not suitable for analysis, with discrepancies resolved among the research team. The exclusion steps were as follows:

First, the exclusion of posts not from Malaysia—During data scraping, longitude and latitude data retained posts located within the coordinates but posted outside of Malaysia, such as parts of Singapore and Thailand. The “location” metadata was therefore used to manually exclude these posts.

Second, the exclusion of irrelevant posts—Irrelevant posts include posts with different definitions (eg, “exercising” your rights), posts not related to health care (eg, religious restrictions on alcohol), indecipherable posts and posts made by bots. Bots were verified by manually checking the user’s profile for any unusual activity patterns that exhibited automated behavior (eg, high frequency of posts without breaks).

#### Data Preprocessing

Before conducting sentiment analysis, data preprocessing was carried out to ensure that the text data was cleaned, transformed, and prepared for analysis.

The model selected for sentiment analysis was the Valence Aware Dictionary and Sentiment Reasoner (VADER) on Python. It is a lexicon-based sentiment analysis tool [25] that is suitable for analyzing social media posts, generating results with high classification success [26]. As VADER is trained for sentiment analysis in English, all posts in Malay and mixed languages were preprocessed and translated into English using the langid and googletrans libraries on Python.

Other data preprocessing steps (tokenization, lower casing of texts, removal of stop words, html links, numbers, punctuations, emojis, and acronyms) were not executed. This is attributed to the unique advantages of VADER, in which assessment scores would account for capitalism, punctuations, emojis, English acronyms (eg, “LOL”), and colloquialisms (eg, “meh”) [27-29].

Following data preprocessing, a word cloud used to provide a visual representation of the words in the overall X dataset.

#### Sentiment Analysis

Computational, lexicon-based sentiment analysis was conducted to determine users’ sentiments. This approach was selected over manual annotations and other types of computational methods as it is cost-effective and does not require training for large datasets. As it relies on a predefined lexicon, it does not require significant computational resources. For general sentiment analysis, lexicon-based approaches often performs well enough to capture the overall sentiment trends in social media data [25].

The posts that have undergone data preprocessing were then analyzed for polarity using VADER. VADER classifies posts into positive, neutral, and negative sentiments. Positive sentiments have a compound score of ≥0.05, neutral sentiments have a score between >−0.05 and<0.05 and negative sentiments have a score of ≤−0.05 [25].

Computer-assisted translation tools may limit the extent of translation in posts that contain local dialects and slang. The translated posts may become indecipherable, causing them to be classified as having “neutral” sentiments. For example, “x” in Malay, which means “no” in English may not have undergone translation, resulting in sentiments not being classified accurately. Following the first round of sentiment analysis, the structures of the translated posts that were unclear and yielded neutral sentiments were manually improved. The sentiment analysis was then re-run to enhance the robustness of sentiment classification and to reduce the inaccurate labeling of posts.

### Manual Content Analysis of Posts With Positive and Negative Sentiments

Sentiment analysis classifies texts according to the emotions conveyed [10]. However, there was no further elaboration on the content and whether users’ perceptions were aligned with recommended health practices.

Therefore, a sample of posts with positive and negative sentiments were randomly selected for manual content analysis. Stratified sampling was conducted by dividing the posts according to the type of lifestyle behavior and sentiment class (positive or negative). For each type of lifestyle behavior and sentiment class, 20% of the total posts for the particular lifestyle behavior were subjected to random selection. This would allow the posts for each lifestyle behavior to have an equal number of positive and negative sentiments. The random sample of posts was generated by using the random number equation in Microsoft Excel relative to the ID number attached to each post. This approach was adopted from a previous content analysis study on X, which also manually coded a random sample of 20% of total posts [30].

A preliminary codebook with 2 categories was developed through discussions among the research team to classify the content of posts and the alignment of users’ perceptions with recommended health practices. This codebook is partially adapted from the codes used by Miller et al [31]. The recommended health practices are based on WHO’s health recommendations [32]. In brief, WHO advocates healthy practices, including abstaining from smoking and alcohol consumption, maintaining a balanced diet, and engaging in regular physical activities. The codes were mutually exclusive. Using the preliminary codebook, 100 posts (25 posts for each type of lifestyle behavior) were independently coded by 2 coders. Interrater reliability was conducted to measure the agreement of each post between both coders. The preliminary codebook was refined until a Cohen kappa score of 0.80 was achieved. The remaining posts were then coded independently using the finalized codebook by both coders.

Table 1 provides a brief description of the codes for the finalized codebook, with a more comprehensive codebook provided in Multimedia Appendix 2.

**Table 1.** Brief description of codes.

Category	Definition
Post content (Topical content dealing with the lifestyle behavior mentioned in the post)	
Self-narrative of current lifestyle behaviors	Narration of self’s current lifestyle behaviors.
Narrative of others’ current lifestyle behaviors	Talked about other people’s current lifestyle behaviors.
Planned action related to lifestyle behaviors	A planned action that will be conducted by the person who wrote the post.
Recommendations related to lifestyle behaviors	A recommendation by the person who wrote the post, providing instruction, advice, or suggestion to others.
Direct question	Direct question used in a post.
General statement	General statement that is not under any of the other categories above.
Alignment of users’ perceptions with recommended health practices (Whether users’ perceptions in posts are aligned with WHO’s health recommendations)	
Aligned with recommended health practices	Users agreed with the conduct of recommended health practices, that included not smoking, avoiding alcohol consumption, maintaining a balanced diet and being physically active.
Not aligned with recommended health practices	Users were not agreeable with the conduct of recommended health practices (eg, consumed oily food, refused to exercise).
Users’ perceptions cannot be defined	The perceptions of the user could not be defined or linked with health practices.

### Data Analysis

Data analysis was performed using descriptive statistics with all variables expressed in frequencies and percentages. The Pearson chi-square test was used to compare the associations between the categorical variables, with *P* values <.05 considered to be statistically significant. IBM SPSS Statistics version 26.0 was used for data analysis.

Findings were also visualized using a word cloud and bar charts. In addition, examples of posts were provided to describe the study findings.

### Ethical Considerations

This study was approved by the Medical Research and Ethics Committee, Ministry of Health Malaysia (NMRR ID-23‐00293-CIM [IIR]) on March 23, 2023, and the Research Ethics Committee, Universiti Kebangsaan Malaysia (UKM PPI/111/8/JEP-2023‐174) on April 13, 2023.

As this study relied solely on publicly available social media data on X and did not involve direct interaction with individuals, informed consent was not applicable. No compensation was offered or provided, as the study did not involve direct participation of human participants.

No identifiable private user information was collected or analyzed. All data used in the analysis were publicly available and did not contain personally identifiable information.

## Results

### Overview of X Dataset

**Figure 2.**
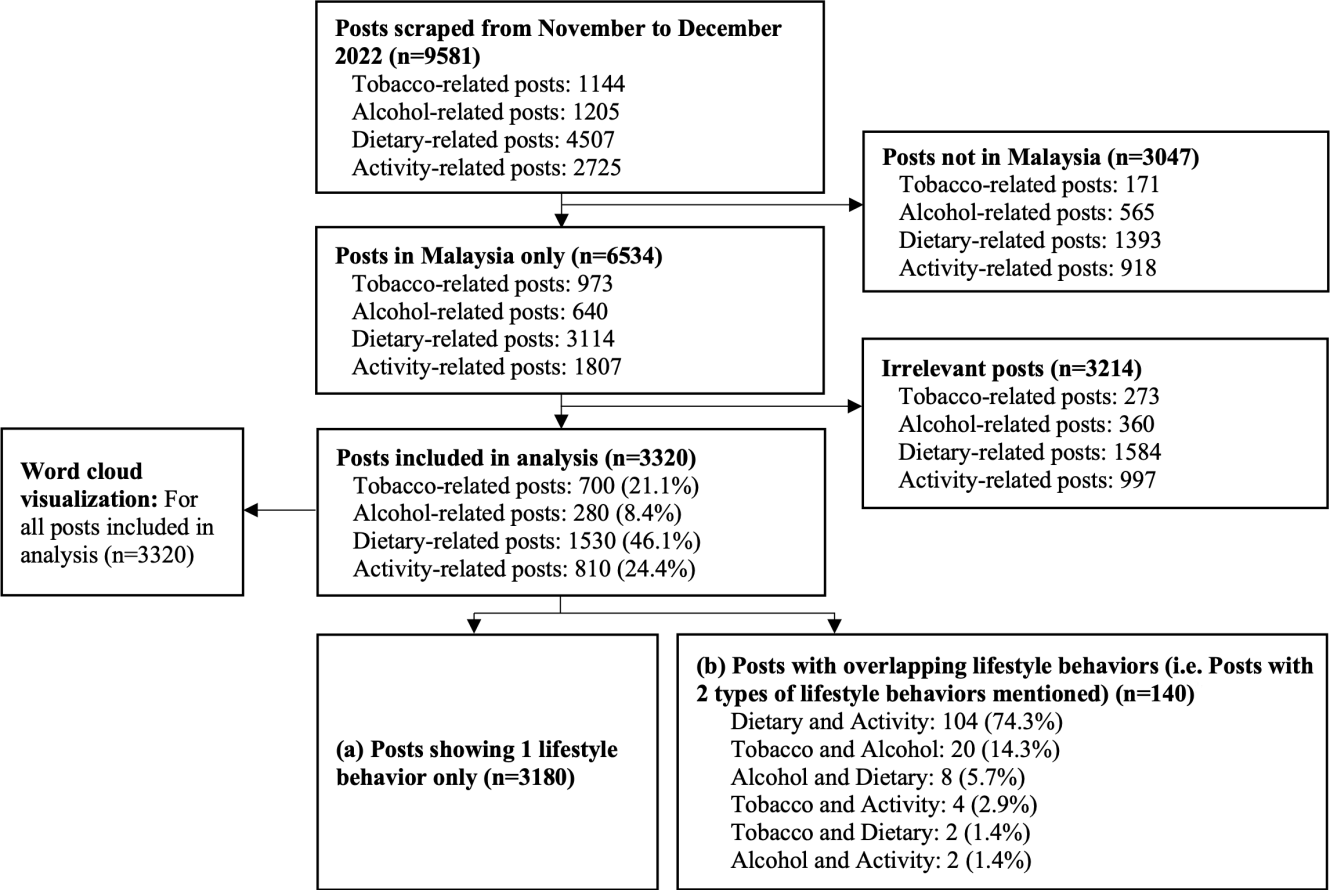
Flowchart of selection of X dataset. Examples of irrelevant posts include (1) tobacco-related posts: gaming-type of posts (eg, smoke mentioned in a game), music band names (eg, Cigarettes After Sex), tweet mentioning terms as a location (eg, Hookah Island, Vape Shop); (2) alcohol-related posts: banning alcohol due to religion with no links to health (eg, at Qatar for the World Cup), sarcasm-based (eg, “you must be drunk,” which translates to “you must be kidding me”); (3) dietary-related posts: nephew in Malay (eg, anak buah), nonhealth posts (eg, fruit on the trees or plants, sugar daddy); (4) activity-related posts: posts mentioning terms as a location (eg, Sports Direct), nonhealth posts (eg, who will be Sports Minister?, “exercising” your rights). With regard to posts overlapping lifestyle behaviors, as data scraping was conducted separately for each lifestyle behavior, a post may appear more than 2 times across different behaviors (eg, the post talks about both smoking and dietary habits).

Figure 2 shows the flowchart of the selection of the X dataset.

A total of 9581 posts were scraped from November to December 2022. Following the exclusion of 3047 posts that were not in Malaysia and 3214 irrelevant posts, 3320 posts across 4 types of lifestyle behaviors were retained for sentiment analysis. Almost half of the posts were dietary-related (1530/3320, 46.1%), followed by activity-related (810/3320, 24.4%) and tobacco-related (700/3320, 21.1%) posts. Alcohol-related posts were present in only one-tenth of the posts (280/3320, 8.4%).

As data scraping was conducted separately for each lifestyle behavior, a post may appear more than two times across different behaviors. Out of the 3320 posts, 3180 (95.8%) posts showed 1 lifestyle behavior only. There were 140 posts with two types of lifestyle of behaviors mentioned, with three-quarters (104/140, 74.3%) of posts mentioning both dietary- and activity-related behaviors.

A word cloud was used to visualize the overall X dataset (n=3320; see Multimedia Appendix 3). Overall, the 5 terms most commonly mentioned by users were “diet,” “rice,” “eat,” “sugar,” and “smoke.”

### Findings From Classification of Sentiments in Posts

Table 2 presents the frequency distribution of sentiment analysis, with examples for each lifestyle behavior provided in Multimedia Appendix 4.

The overall percentage of positive sentiments almost doubled that of negative sentiments (1874/3320, 56.5%) vs (1027/3320, 30.9%). Results have shown a significant association between sentiment class and lifestyle behaviors (*χ*^2^_6_=67.64; *P*<.001), with positive sentiments being higher than negative sentiments for all lifestyle behaviors. The trends for dietary- and activity-related posts were similar, with both showing approximate percentages of 60% for positive sentiments and 27% for negative sentiments. This was followed by alcohol-related posts with positive sentiments of 54.7% (153/280). Less than half of tobacco-related posts (314/700, 44.9%) had positive sentiments, with the percentage differences between sentiment classes slightly below 2%.

**Table 2.** Frequency distribution of sentiment analysis before and after structure improvement of posts using Valence Aware Dictionary and Sentiment Reasoner (VADER; n=3320).

Lifestyle behaviors	Sentiment count before structure improvement of posts, n (%)			Sentiment count after structure improvement of posts, n (%)^a^			Total, n
Positive	Neutral	Negative	Positive	Neutral	Negative
Tobacco-related posts	254 (36.3)	197 (28.1)	249 (35.6)	314 (44.9)	84 (12)	302 (43.1)	700
Alcohol-related posts	120 (42.9)	95 (33.9)	65 (23.2)	153 (54.7)	41 (14.6)	86 (30.7)	280
Dietary-related posts	724 (47.3)	455 (29.7)	351 (23)	916 (59.9)	197 (12.9)	417 (27.2)	1530
Activity-related posts	378 (46.7)	246 (30.4)	186 (22.9)	491 (60.6)	97 (12)	222 (27.4)	810
Total	—^b^	—	—	1874 (56.5)	419 (12.6)	1027 (30.9)	3320

a Sentiment count after structure improvement of posts were used for analysis. A Pearson chi-square test was conducted to test the associations between sentiment class and lifestyle behaviors (*χ*2_6_=67.64, *P*<.001).

b Not applicable.

### Findings From Manual Content Analysis of Posts

A total of 1328 posts with an equal number of positive and negative sentiments for each lifestyle behavior were selected for manual content analysis. They comprised of 280 tobacco-related posts (140 posts for each sentiment class), 112 alcohol-related posts (56 posts for each sentiment class), 612 dietary-related posts (306 posts for each sentiment class), and 324 activity-related posts (162 posts for each sentiment class).

Prior to the manual content analysis of all 1328 posts, 100 posts were first subjected to interrater reliability testing. The Cohen kappa scores for both categories of post content and the alignment of users’ perceptions with recommended health practices were 0.807 and 0.801, respectively.

The frequency of posts is tabulated in Table 3. Overall, the content with the 3 highest number of posts were self-narratives of current lifestyle behaviors (492/1328, 37%), general statements (203/1328, 15.3%) and planned actions (196/1328, 14.8%). Self-narratives were the most popular content for all types of lifestyle behaviors except for tobacco-related posts, in which the majority were narratives of others’ current behaviors (96/280, 34.3%). Question-based posts were the least popular content for tobacco-, alcohol-, and activity-related posts, with less than 10% present in all types. Users’ perceptions in more than half of the posts were aligned with recommended health practices (769/1328, 57.9%). Similar proportions were observed for all types of lifestyle behaviors except for alcohol-related posts, in which posts not aligned with recommended health practices were double those aligned with recommended health practices (63/112, 56.3% vs 33/112, 29.4%).

**Figure 3.**
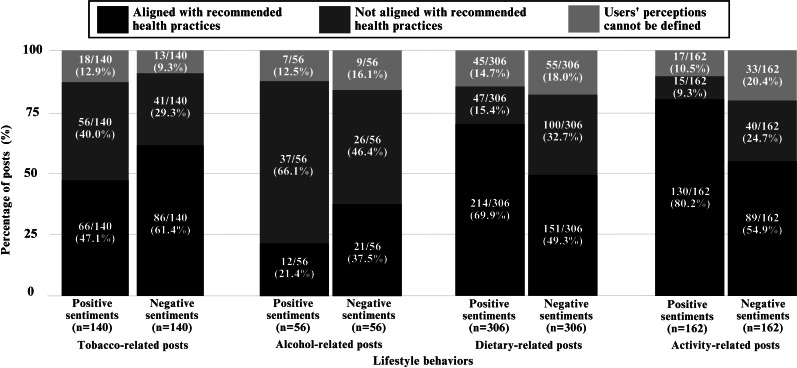
Stacked bar charts of alignment of users’ perceptions with recommended health practices stratified according to sentiment classification. A Pearson chi-square test was conducted for each lifestyle behavior to test the associations between sentiment class and alignment of users’ perceptions with recommended health practices (tobacco: *χ*^2^_2_=5.76, *P*=.06; alcohol: *χ*^2^_2_=4.62, *P*=.10; dietary: *χ*^2^_2_=30.98, *P<*.001; activity: *χ*^2^_2_=24.16, *P<*.001).

Figure 3 shows the frequency of posts that demonstrated the alignment of users’ perceptions with recommended health practices according to the sentiment classification. A total of 3 main findings were observed. First, in dietary- and activity-related posts, significant associations between sentiment class and alignment of users’ perceptions with recommended health practices were observed (*χ*^2^_2_=30.98, *P*<.001 and *χ*^2^_2_=24.16, *P*<.001; respectively). In both positive and negative sentiment classes, the percentages of posts aligned with recommended health practices were significantly higher than those not aligned with recommended health practices and those with undefined user perceptions, with percentages ranging from 49.3% to 80.2%. Posts with positive sentiments that aligned with recommended healthy practices showcased users’ optimism to stay healthy (eg, “I’m ready to cut sugar. Let’s go” [D-919-positive]). Meanwhile, negative sentiments that aligned with recommended healthy practices highlighted users’ worries to stay healthy (eg, “Feel the weight.. rise suddenly. Sad. Have to fix it” [P-522-negative]).

Second, in tobacco-related posts, there was no significant association between sentiment class and alignment of users’ perceptions with recommended health practices (*χ*^2^_2_=5.76; *P*=.06). Among posts with positive sentiments, the percentage of posts that aligned with recommended health practices was similar to those not aligned with recommended health practices, with a percentage difference of 7.1%. When users posted about tobacco with positive emotions, the likelihood of their perceptions aligning with the recommended health practices of not smoking (eg, “Please pray that I can stop smoking…” [T-563-positive]) or aligning with hazardous smoking practices (eg, “My kind of chill with cigar” [T-412-positive]) were similar. Despite the lack of significant association, the percentage of posts with negative sentiments that aligned with recommended health practices was noticeably higher than those that did not align with recommended health practices (86/140, 61.4% vs 41/140, 29.3%).

Third, a lack of significant association between sentiment class and alignment of users’ perceptions with recommended health practices was also observed in alcohol-related posts (*χ*^2^_2_=4.62; *P*=.10). Although the findings were not statistically significant, the percentage of posts not aligned with recommended health practices was higher than those aligned with recommended health practices for both positive (37/56, 66.1% vs 12/56, 21.4%) and negative (26/56, 46.4% vs 21/56, 37.5%) sentiment classes. Positive sentiments leading to alcohol consumption included celebratory posts (eg, “I’m gonna have so much wine this weekend” [A-55-positive]), whereas negative sentiments involved users coping with worries (“I am going to drown my sorrows in alcohol and pick things back up tomorrow” [A-198-negative]). Examples of posts selected for manual content analysis are provided in Multimedia Appendix 5.

**Table 3.** Frequency of posts according to post content and alignment of users’ perceptions with recommended health practices (n=1328).

Category	Lifestyle behaviors				Total posts (N=1328)
	Tobacco-related (n=280)	Alcohol-related (n=112)	Dietary-related (n=612)	Activity-related (n=324)	
Post content, n (%)					
Self-narrative of current lifestyle behaviors	70 (25)	36 (32.1)	243 (39.7)	143 (44.1)	492 (37)
Narrative of others’ current lifestyle	96 (34.3)	17 (15.2)	40 (6.5)	22 (6.8)	175 (13.2)
Planned action related to lifestyle behaviors	26 (9.3)	15 (13.4)	97 (15.8)	58 (17.9)	196 (14.8)
Recommendations related to lifestyle behaviors	35 (12.5)	13 (11.6)	83 (13.6)	46 (14.2)	177 (13.3)
Direct question	17 (6.1)	6 (5.4)	45 (7.4)	17 (5.3)	85 (6.4)
General statement	36 (12.8)	25 (22.3)	104 (17)	38 (11.7)	203 (15.3)
Alignment of users’ perceptions with recommended health practices, n (%)					
Aligned with recommended health practices	152 (54.3)	33 (29.4)	365 (59.7)	219 (67.6)	769 (57.9)
Not aligned with recommended health practices	97 (34.6)	63 (56.3)	147 (24)	55 (17)	362 (27.3)
Users’ perceptions cannot be defined	31 (11.1)	16 (14.3)	100 (16.3)	50 (15.4)	197 (14.8)

## Discussion

### Overview

To the best of our knowledge, this is the first study in the region that examined discussions on X across multiple lifestyle behaviors. This study is also the first of its kind that used dual approaches of lexicon-based sentiment analysis and manual content analysis of posts to examine users’ sentiments, post content and the alignment of users’ perceptions with recommended health practices. Positive sentiments were significantly higher than negative sentiments for all 4 lifestyle behaviors. In dietary- and activity-related posts, users exhibited twice as many positive sentiments as negative ones. The majority of the sampled posts were self-narratives of current lifestyle behaviors. More than half of the sampled tobacco-, dietary-, and activity-related posts were aligned with WHO’s recommended health practices, with contrasting results in alcohol-related posts.

### Principal Findings

Data scraping has shown that dietary-related topics were the lifestyle behaviors most frequently discussed. The usage of a more extensive set of search terms in scraping dietary-related posts covered a variety of nutrition-based topics involving individuals across all age groups. This resulted in dietary-related discourses among young children and adolescents (eg, formula milk, vegetables, and fruits consumption). Users frequently mentioned “rice,” which is attributed to Malaysians’ staple diet, with the average Malaysian adult consuming 82.3 kilograms of rice annually [33]. In contrast, alcohol-related topics were the least discussed lifestyle behaviors among users. Alcohol-related discussions in Malaysia were largely anchored on themes related to cultural and religious beliefs. Alcohol consumption among Malaysians is generally lower as behaviors are influenced by compartmentalization among the three main races in Malaysia [34]. Malays who are Muslims are not allowed to consume alcohol as it is forbidden in Islam [35], whereas no restrictions were imposed on the Chinese and Indian communities [34]. The prohibition of alcohol consumption in certain communities was hypothesized as one of the reasons for the lower frequency of alcohol-related posts, compared to other lifestyle behaviors.

Findings indicate that positive sentiments significantly outweighed negative sentiments for all lifestyle behaviors. In dietary- and activity-related posts, positive sentiments were found to be twice as many as negative sentiments, which is consistent with the sentiment analysis findings of Shaw et al [36] who analyzed over 1.5 million posts on dietary and exercise topics. In our study, more than half of the sampled posts were either self-narratives or planned actions for self-implementation. It could be postulated that the posts with positive sentiments were driven by self-determination theory (SDT), a comprehensive theory of human motivation and personality that focuses on individuals’ intrinsic tendencies for growth. SDT assumes the importance of autonomous motivation, which is a type of self-emanating motivation that is consistent with users’ innate values to engage in behaviors or pursue a goal [37,38]. The field of autonomous motivation has been extensively studied in the context of dietary and exercise lifestyle behaviors [39-41]. Individuals who are autonomously motivated have a sense of self-control over their actions (eg, choose to exercise regularly), leading to an increase in positive sentiments, personal fulfilment and enjoyment in the actions pursued [40].

In tobacco-related posts, positive sentiments were found to be higher than negative sentiments, albeit with a small percentage difference. This suggests that in tobacco-related discourses, users tend to either feel positive emotions (eg, satisfaction, happiness, and trust) or negative emotions (eg, dissatisfaction, unhappiness, and worry). Mixed sentiments were found to be prevalent in discussions related to vaping among both the scientific community [42], and the general public [43]. In Malaysia, the Health Ministry has proposed the Generational End Game plan, which would ban tobacco sales for those born after 2005. The bill was first tabled at the country’s parliamentary discussions in July 2022 and has yet to be finalized at the time when the social media posts were scraped from X [44]. Such uncertainties towards health policy changes have generated both positive and negative reactions, with the issue being debated constantly throughout the year. Users either praised the government’s efforts to mitigate smoking behaviors or expressed concerns about such “untested” plans [45]. The negative reactions may also stemmed from users’ awareness of the adverse effects of smoking, with over 90% of male lung cancer patients in Malaysia having a significant history of smoking [46]. In addition, 3500 out of 10,000 annual deaths were linked to smoking [47].

As discussed, the predominance of self-narratives in posts related to diet, physical activity and alcohol consumption is likely due to users’ autonomous motivation and self-awareness to perform a behavior. Conversely, most posts related to tobacco were found to be linked to narratives of other users. As tobacco smoking has been associated with social stigmatization due to its negative health impact on others, users may have been more reluctant to post from a first-person perspective. Instead, users opt to openly discuss the smoking habits of others. In addition, sharing experiences in a third-person perspective may be preferred by users to maintain anonymity. During the 20th century, smokers were often viewed as “mysterious” or “cool,” but this social status has slowly diminished over the past two decades [48,49]. In Malaysia, this was propelled by smoking reduction strategies, such as the ban on smoking in public eateries implemented in 2019, that socially impacted users’ impression toward smoking [50].

In recent years, there has been a growing trend of health influencers using online platforms to actively share their dietary and fitness regimens. Previous studies have shown that social media users who were exposed to this information delivered by health influencers as well as content from other social media users, were more likely to be receptive to adopting healthy practices, such as maintaining a balanced diet and being physically active [51-53]. The results were consistent with the findings of this study, which showed a significantly higher percentage of dietary- and activity-related posts by social media users that were aligned with recommended health practices. Nevertheless, HCPs must remain active in advocating positive lifestyle behaviors on social media. Although almost one-fifth of posts for these two lifestyle behaviors were on planned actions, this may not always translate into actions by the population. This is a caveat of much research that relies on social media or self-reported data on social media. It is often unclear whether individuals actually follow through on what they post about, highlighting the intention-behavior gap [54]. Findings from the Malaysian National Health and Morbidity Survey (NHMS) survey conducted in 2023 have shown that the actual adoption of healthy practices was still lacking among the Malaysian public. Almost 95.1% Malaysian adults did not meet the recommended daily intake of fruits and vegetables, consuming only two servings of fruit or vegetables daily instead of the recommended five servings daily [3] The prevalence of physical inactivity among Malaysian adults was at 29.9% [3], which was also considerably higher than other Asian countries, including China and India [55,56].

There was a lack of significant association between sentiment class and alignment with recommended health practices in both tobacco- and alcohol-related posts. Despite the smaller number of sampled alcohol-related posts, it is interesting to note that users’ perceptions with recommended health practices had contrasting outcomes compared to the other three lifestyle behaviors. In more than half of the alcohol-related posts with positive and negative sentiments, users’ perceptions were not aligned with recommended health practices. Most users perceived alcohol consumption as a casual and an affordable social activity and did not acknowledge the potential health risks involved. A survey conducted in Thailand, a country of similar income setting, had previously mentioned the popularity of alcohol being a social activity among urban communities [57]. In Malaysia, alcoholic beverages were available for purchase at neighborhood convenience stores, which allowed for easy purchases of takeaway alcohol [35]. This further downplayed users’ awareness of the negative consequences of alcohol consumption [58].

Assessment of posts made by social media users on X allows HCPs to identify priority areas for social media-based health information delivery on this platform. As most alcohol-related posts do not align with health recommendations, it is postulated that greater emphasis should be placed on strategies to limit alcohol consumption among users in Malaysia. The WHO has proposed collaborative efforts with HCPs and journalists to improve targeted public health messaging to the public. A guide was recently developed for journalists to facilitate media reporting to communities on the harms of alcohol consumption [58]. While the other 3 lifestyle behaviors were mostly aligned with recommended health practices, it remains essential for HCPs to continuously deliver information advocating healthy behaviors. Online approaches allow HCPs to deliver information beyond geographic barriers, reaching a wider audience in diverse community settings. Therefore, health information can be adopted by users in countries with similar cultural beliefs, including countries within the Southeast Asia region.

### Strengths and Limitations

The strengths of this study included its comprehensive coverage of 4 lifestyle behaviors aimed at reducing the 4 key modifiable risk factors under WHO’s health priority [4]. This allowed for the simultaneous analysis of posts across different lifestyle behaviors. Unlike most studies that focus on global contexts, this research uniquely focused on the Malaysian context. It provides insights into the cultural and social dynamics that influence discussions around lifestyle behaviors in this specific region. Notably, the inclusion of alcohol-related posts in analysis shed light on culturally and socially nuanced discussions within the region. This is particularly valuable in regions like Malaysia, where religious and cultural factors strongly influence alcohol consumption. In addition, the retrospective examination of social media posts utilized approaches of lexicon-based sentiment analysis and manual content analysis. The dual approach provided real-time and spontaneous insights into users’ opinions on lifestyle behaviors while addressing limitations of single-method studies. Findings from this study could assist HCPs in prioritizing the delivery of region-specific health information through social media.

Nevertheless, a few limitations should be considered. First, potential bias may exist during the selection of social media dataset. In self-selection bias, users who choose to share their opinions on social media may not represent the broader population. The study may be subjected to data selection bias as it included only social media posts in Malay and English, excluding other spoken languages in Malaysia, such as Chinese and Tamil. Nevertheless, sentiment analysis studies on X in Malaysia have largely concentrated on data scraped in Malay and English [59,60]. Demographic bias may be present due to the overrepresentation or underrepresentation of certain groups on X. For instance, the majority of users in Malaysia who post on social media are aged between 25 and 34 years old [9]. The limitations in the availability of metadata on X also prevented the collection of demographic data such as age, gender and race, as most users did not disclose this information in their profiles. Population bias may occur in geotagged posts utilizing longitude and latitude metadata. Previously literature has indicated that only 1% of users would geotag their location in posts [61]. Nevertheless, this is the most effective method to scrape posts that are published within a specific location.

Second, there are limitations in the study design and methods used for sentiment analysis and manual content analysis. The study is cross-sectional in nature and provides a snapshot of discussions at a specific time. Therefore, temporal bias may exist, making it challenging to track changes in sentiments or behaviors over time. In addition, posts that were collected for sentiment analysis and manual content analysis over two consecutive months may not accurately reflect year-round sentiments and discussions, as findings may vary due to the presence of health-related events occurring at certain times of the year. The events may include a change in legislations, prominent public health campaigns or disease outbreaks. The quality of the dataset was ensured by verifying that there were no notable health-related occurrences between November and December 2022. In addition, previous studies analyzing users’ sentiments and content have similarly explored health data over two consecutive months [23,24]. The manual content analysis of social media posts can be time-consuming due to the involvement of large datasets, therefore, only 20% of the total posts were randomly selected using stratified sampling. This percentage was previously utilized in a content analysis study by Mathieson et al [30]. While analyzing the full dataset would provide more comprehensive findings, the randomized sample offers a reliable snapshot for identifying the thematic content without the substantial time and resource demands of manual analysis for the entire dataset. Furthermore, prior to sentiment analysis, the computer-assisted translation of posts from Malay to English may have led to inaccuracies due to the usage of local dialects, sarcasm or slangs. To enhance sentiment labeling, the structures of translated posts with neutral sentiments that were unclear were manually refined, and sentiment analysis was repeated.

Third, the study results should be interpreted with caution, regarding posts on alcohol consumption due to the smaller sample size of 112 posts. A power analysis indicated that this sample size is adequate for detecting effects, with a power of 0.82. Furthermore, we acknowledge the presence of potential interactions in posts with overlapping lifestyle behaviors (eg, a post that talks about diet and physical activity). In sentiment analysis, the conduct of Pearson chi-square tests also did not account for potential confounding factors or interactions in posts with overlapping lifestyle behaviors. To account for this limitation, we compared the proportions between sentiment count for posts showing 1 lifestyle behavior only (n=3180), and sentiment count for posts across 4 types of lifestyle behaviors (n=3320). The proportions of sentiment counts for both were similar to each other. In addition, while many of the Pearson chi-square associations were significant, these may not imply causality and thus may not inform categorically that the observed sentiments result in practicing different lifestyle behaviors or the direction of the relationship.

### Implications and Further Research

The findings from this study could help HCPs to prioritize the delivery of health information on lifestyle behaviors using social media tailored to the targeted region, which is Malaysia. Given the low number of alcohol-related posts by social media users in Malaysia, HCPs could focus on initiating positive discussions around this topic to raise awareness about the harmful effects of alcohol consumption. In addition, most of the alcohol-related posts made by social media users were not aligned with recommended health practices. There is an increased need for HCPs to emphasize on limiting and stopping alcohol consumption, while also acknowledging that the users’ attitudes towards alcohol consumption may still vary among different religions in Malaysia. Health advocacy for positive lifestyle behaviors on social media should continue for the other three lifestyle behaviors.

Further research could be proposed to explore the opinions of social media users toward lifestyle behaviors in Malaysia. First, despite the statistical significance observed in the associations between sentiment classification and lifestyle behaviors, the percentage difference between both sentiment classes in tobacco-related posts was small. Therefore, it would be interesting to investigate whether tobacco sentiments would vary over time. We may want to further track sentiments by time series analysis to explore changes in users’ emotions towards tobacco across a time period. The tracking of real-time sentiments across a time period was previously conducted in a review examining public health data on X that included posts on alcohol consumption [62]. In addition, since posts are scraped based on location metadata, future studies could leverage on this data to explore the relationship between the prevalence of specific lifestyle behaviors in certain locations (eg, urban areas in Malaysia) and the intensity of lifestyle behavior-related discussions on social media. A similar study has previously been conducted in the United States; therefore, conducting such studies in the Malaysian context would be beneficial [63].

Second, the majority of posts involved content related to self-narratives of lifestyle behaviors. These self-narratives outlined X’s roles as a microblog for users to freely express the behaviors they practice from a first-person perspective. As self-narratives encompass a broad and generalized category, it may be beneficial to conduct a more detailed examination of posts that only described users’ self-narratives. This in-depth analysis would provide insights into the specific themes commonly discussed by users from a first-person perspective. In addition, the examination of posts could be extended to other lifestyle behaviors such as sleep patterns, which is particularly relevant as active social media users are mainly adolescents and young adults who are commonly affected by sleep-related issues [64].

Third, this study was conducted on the microblogging platform X. It is also important to examine social media posts made by users on other platforms, such as Facebook. Future research is proposed to analyze the sentiments and content of posts on these platforms. Audience demographics can vary across these platforms. For instance, younger millennials may be more active on X, whereas Facebook often attracts a slightly older audience [65,66]. Comparing our study findings with those obtained from Facebook could help HCPs to deliver health messages that suit the audiences of different social media platforms.

Fourth, our study emphasizes accessibility and simplicity in data visualization and reporting to effectively communicate findings to a diverse audience, including non-technical stakeholders such as HCPs, public health practitioners and policymakers. To achieve this, we employed techniques like word clouds, which provide a visually appealing representation of frequently mentioned terms in the dataset, and lexicon-based sentiment analysis, which is straightforward to implement as it does not require additional labeled data or extensive training. We recognize the potential value of more advanced methods and suggest exploring these techniques in future studies related to the conduct of in-depth text analysis. These may include approaches like topic modeling or keyword co-occurrence analysis to summarize text data through word groups, as well as training machine learning models such as support vector machines or Naïve Bayes to classify sentiments. Furthermore, hybrid methods of sentiment analysis could be explored by integrating machine learning models with lexicon-based approaches. These combined models can then be assessed for accuracy and robustness through comparative analysis. Similar studies have been conducted previously in both health and non-health posts [67,68].

### Conclusion

In conclusion, the incorporation of lexicon-based sentiment analysis holds significance as it enabled the use of large amounts of data to capture users’ emotions whilst posting on lifestyle behaviors. Positive sentiments were significantly expressed in posts for all lifestyle behaviors. Nevertheless, there was a small percentage difference observed in tobacco-related posts, indicating a more varied sentiment among users. Most of the posts showed users’ own narratives and planned actions towards the conduct of a behavior. As the majority of alcohol-related discussions were not aligned with recommended health practices, this reflects the need for individual HCPs and health organizations to increase their delivery of health information pertaining to alcohol consumption on social media platforms. It is also equally important for HCPs to continue providing health information on other lifestyle behaviors to social media users, while monitoring ongoing discussions by users on social media.

## Supplementary material

10.2196/65835 Multimedia Appendix 1 List of keywords for data scraping of posts.

10.2196/65835 Multimedia Appendix 2 Codebook.

10.2196/65835 Multimedia Appendix 3 Word cloud representation of overall X dataset (n=3320).

10.2196/65835 Multimedia Appendix 4 Examples of posts with positive and negative sentiments according to each lifestyle behavior.

10.2196/65835 Multimedia Appendix 5 Example of posts selected for manual content analysis.

## Abbreviations

HCP: health care professional
NCD: noncommunicable disease
NHMS: National Health and Morbidity Survey
SDT: self-determination theory
VADER: Valence Aware Dictionary and Sentiment Reasoner
WHO: World Health Organization
