# Supplementary material for: Exploring Social Media Posts on Lifestyle Behaviors: Sentiment and Content Analysis
Source: JMIR Infodemiology. 2025 Jun 25;5:e65835. doi: 10.2196/65835 (PMC12221188; doi:10.2196/65835)
Supplement: Multimedia Appendix 1 [file infodemiology-v5-e65835-s001.docx]

**Multimedia Appendix 1.** List of keywords for data scraping of posts.

| - Timeline from November 2022 to December 2022. - Malay and / or English posts by social media users, geographically within Malaysia. - Scraping of data conducted on 16^th^ April 2023. | | |
| --- | --- | --- |
| **No** | **Lifestyle behaviors** | **Keywords in English and Malay** |
| 1 | Tobacco-related posts | smoke OR smoking OR smoker OR nicotine OR tobacco OR cigar OR cigars OR cigarette OR cigarettes OR vape OR vaping OR shisha OR hookah OR rokok OR merokok OR perokok OR nikotin OR tembakau |
| 2 | Alcohol-related posts | alcohol OR alcoholic OR liquor OR liqueur OR spirits OR beer OR tuak OR toddy OR samsu OR tapai OR wine OR gin OR vodka OR whiskey OR tequila OR rum OR brandy OR alkohol OR "minuman keras" OR arak |
| 3 | Dietary-related posts | diet OR pemakanan OR "balanced diet" OR "healthy diet" OR "eat healthy" OR fruit OR fruits OR vegetable OR vegetables OR rice OR cereal OR grains OR "plain water" OR "diet seimbang" OR "makan seimbang" OR "makanan seimbang" OR "pemakanan seimbang" OR buah OR sayur OR "nasi putih" OR bijirin OR "air kosong" OR "unhealthy diet" OR "eat unhealthy" OR "junk food" OR "fast food" OR "soft drink" OR "soft drinks" OR "pemakanan tidak sihat" OR "pemakanan tidak seimbang" OR "makanan ringan" OR "makanan segera" OR "minuman berkarbonat" OR "saturated fat" OR sugar OR salt OR "lemak tepu" OR gula OR garam |
| 4 | Activity-related posts | "body mass index" OR BMI OR "body weight" OR "indeks jisim" OR "berat badan" OR exercise OR exercises OR exercising OR "physical activity" OR sport OR sports OR workout OR senaman OR bersenam OR "aktiviti fizikal" OR sukan OR bersukan OR overweight OR obese OR obesity OR sedentary OR "physical inactivity" OR "inactive" OR "berat badan lebih" OR obes OR obesiti OR sedentari OR "aktiviti tidak sihat" OR "tidak aktif" |
