# Supplementary material for: Exploring Social Media Posts on Lifestyle Behaviors: Sentiment and Content Analysis
Source: JMIR Infodemiology. 2025 Jun 25;5:e65835. doi: 10.2196/65835 (PMC12221188; doi:10.2196/65835)
Supplement: Multimedia Appendix 2 [file infodemiology-v5-e65835-s002.docx]

**Multimedia Appendix 2.** Codebook.

**Table 2.1.** Post content **(**Topical content dealing with the lifestyle behavior mentioned in the post)^a^.

| **No** | **Coding** | **Definition** |
| --- | --- | --- |
| 1 | Self-narrative of current lifestyle behaviors | - **Main definition:** Narration of self’s current lifestyle behaviors. - “Self” referred to the person who wrote the post. - Usually have the usage of pronouns such as ‘I’, ‘me’, ‘myself’ or its Malay derivatives. - Sometimes no pronouns were present but if the content of the post was a narration of the person who wrote the post, it was classified in this category. - Context of post referred to a lifestyle behavior that *has already* happened or *currently* still happening. - **Example:** “I have been smoking for 10 years, my life is still great.” |
| 2 | Narrative of other’s current lifestyle behaviors | - **Main definition:** Talked about other people’s current lifestyle behaviors. - “Other people” referred to people who were not involved in writing the post (e.g. friends, family members, acquaintances or public figures). - Usually have the usage of pronouns such as ‘my sister,’ ‘my grandfather,’ ‘they’ etc. - Sometimes no pronouns were present but if the content of the post was a narration of someone who did not write the post, it was classified in this coding. - Context of post referred to a lifestyle behavior that *has already* happened or *currently* still happening. - **Example:** “He really doesn’t listen and continues to drink so much alcohol daily.” |
| 3 | Planned action related to lifestyle behaviors | - **Main definition:** A planned action that will be conducted by the person who wrote the post. - The planned action was either a healthy action or an unhealthy action related to lifestyle behaviors. - The planned action has not happened yet, at the time post was made. - May have usage of terms such as “wish,” “want,” “plan," “next week,” “next year.” - Planned action was either:  1. *A direct action:* Aimed at changes in lifestyle behaviors directly (e.g. need to start diet, need to smoke more, want to exercise less). 2. *An indirect action:* Aimed at changes in lifestyle behaviors indirectly (e.g. start buying more alcohol over the weekend, start going to places that allow smoking).  - The current status of planned action was either:  1. *Initiation of planned action:* (e.g. need to start diet, want to buy cigarettes to try smoking, need to diet). 2. *An increase or decrease in the frequency in planned action:* (e.g. need to smoke more, want to exercise less). |

**Multimedia Appendix 2.** Codebook. (continued)

**Table 2.1.** Post content **(**Topical content dealing with the lifestyle behavior mentioned in the post)^a^. (continued)

| **No** | **Coding** | **Definition** |
| --- | --- | --- |
| 4 | Recommendation related to lifestyle behaviors | - **Main definition:** A recommendation by the person who wrote the post, providing instruction, advice or suggestion to others. - The recommendation was either a healthy recommendation or an unhealthy recommendation related to lifestyle behaviors. - Recommendations in the form of:  1. *Instruction:* May have usage of terms such as “please ensure,” please make sure,” “please do it.” 2. *Advice:* May have usage of terms such as “you should,” “try to follow,” “do or don’t <insert an action>,” “go <insert action>.” 3. *Suggestion:* May have usage of terms such as “suggest,” “propose” 4. Additionally, may be a direct post aiming to promote lifestyle behaviors (e.g. apple cider has a lot of benefits to health, buy fast food to save time).  - A recommendation was either a:  1. *A direct recommendation:* Aimed at direct changes in lifestyle behaviors (e.g. you should drink more alcohol as it keeps you high, don’t smoke) 2. *An indirect recommendation:* Aimed at indirect changes in lifestyle behaviors (e.g. the government should legalize vape, get medical card if you smoke, read your daily verses before you smoke)  - The recommendation may be in the form of a question. In this case, they were placed under this coding instead of “direct question.” For example, “why not just ban all youngsters from vaping?” which suggested the banning of youngsters from vaping. |
| 5 | Direct question | - **Main definition:** Direct question used in a post. - The content of the post was a question on its own and expected an answer either by:  1. The person reading the post (e.g. Anyone want to workout this Sunday?) 2. The person whom the post is directed at (e.g. Have you had your lunch on time?) 3. The person who posted the original post (e.g. Why am I still smoking?)  - Questions were purely about asking for an answer that may come with or without the “question mark” (?) symbol (e.g. Should I walk to the sports center). - Recommendations in the form of questions were placed in “recommendations related to lifestyle behaviors.” instead of “direct question” (e.g. Why not just ban all youngsters from vaping?). |
| 6 | General statement | - **Main definition:** General statement that was not under any of the other categories above. - A general statement referred to:  1. Posts that utilized nouns alone (e.g. fried rice 3 plates, yoga, smoke 10 cigarettes). 2. Posts that described a situation (e.g. there’s no alcohol here, people are throwing their cigarette buds everywhere). 3. Sentences that were not complete (e.g. if he is obese, yay gula manis). 4. Did not meet the criteria of other categories (e.g. People have been calculating BMI formula for years, 70-100kg body weight is a bit inappropriate, ½ liter of beer is RM200).  - Most of the posts under “general statement” had little to no links with the person who posted the post. |

**^a^**Codes were mutually-exclusive. For posts were able to be categorized into 2 codes, the coding was based on the part of the post that was directly linked to the lifestyle behavior mentioned. For example, “Youngsters these days might think that drinking alcohol is good but I am honestly not okay with it.” The lifestyle behavior of alcohol consumption is linked to others (i.e. youngsters), therefore, the selected coding was “narration of other’s current lifestyle behaviors.”

**Multimedia Appendix 2.** Codebook. (continued)

**Table 2.2** Alignment of users’ perceptions with recommended health practices (Whether users’ perceptions in posts were aligned with WHO’s health recommendations).

| **No** | **Coding** | **Definition** |
| --- | --- | --- |
| 1 | Aligned with recommended health practices | - **Main definition:** Users agreed with the conduct of recommended health practices, that included not smoking, avoiding alcohol consumption, maintaining a balanced diet and being physically active. - Coding was based on users’ perceptions in the posts. - **Users’ perceptions were defined in 3 different situations:**  1. *Post mentioned the person who posted the content* ***only*** *(with or without the use of pronouns):* Users’ perceptions were based on the person who posted the content (e.g. “I lost 20 kg last year by working out, I feel at ease now,” showed alignment with recommended health practices). 2. *Post mentioned a third party* ***only*** *(with or without the use of pronouns):* Users’ perceptions were based on the person mentioned in the post (e.g. “My brothers don’t smoke and vape” and “He has been eating more vegetables” showed alignment with recommended health practices). 3. *Post mentioned* ***both*** *the person who posted the content and a third party (with or without the use of pronouns):* Users’ perceptions were based on the person who posted the content (e.g. “Youngsters these days might think that drinking alcohol is good but I am honestly not okay with it” and “Can’t believe there are some silly people who chose to smoke at the children’s playground” showed alignment with recommended health practices). |
| 2 | Not aligned with recommended health practices | - **Main definition:** Users were not agreeable with the conduct of recommended health practices (e.g. consumed oily food, refused to exercise). - Coding was based on users’ perceptions in the posts. - **Users’ perceptions were defined in 3 different situations:**  1. *Post mentioned the person who posted the content* ***only*** *(with or without the use of pronouns):* Users’ perceptions were based on the person who posted the content (e.g. **“**Happy that my diet failed. I am not eating vegetables” did not show alignment with recommended health practices.) 2. *Post mentioned a third party* ***only*** *(with or without the use of pronouns):* Users’ perceptions were based on the person mentioned in the post (e.g. “My brothers smoke 10 boxes of cigarettes a day” did not show alignment with recommended health practices). 3. *Post mentioned* ***both*** *the person who posted the content and a third party (with or without the use of pronouns):* Users’ perceptions were based on the person who posted the content (e.g. “Why is everyone not smoking? I really need to light a cigarette now” and “The way they are smoking in their house, should be okay for their kids still” does not show alignment with recommended health practices). |
| 3 | Users’ perceptions cannot be defined | - **Main definition:** The perceptions of the user could not be defined or linked with health practices. - Most posts labelled in “general statements” were placed in this coding. - However, other posts were also likely to be in this coding (e.g. “nowadays I have difficulties in gaining weight,” “what does it mean by smoking?,“ “diet can ruin me anytime.” We were not able to define if users’ perceptions in these posts were aligned or not aligned with recommended health practices. |
