# Supplementary figures and images for: Exploring Social Media Posts on Lifestyle Behaviors: Sentiment and Content Analysis
Source: JMIR Infodemiology. 2025 Jun 25;5:e65835. doi: 10.2196/65835 (PMC12221188; doi:10.2196/65835)

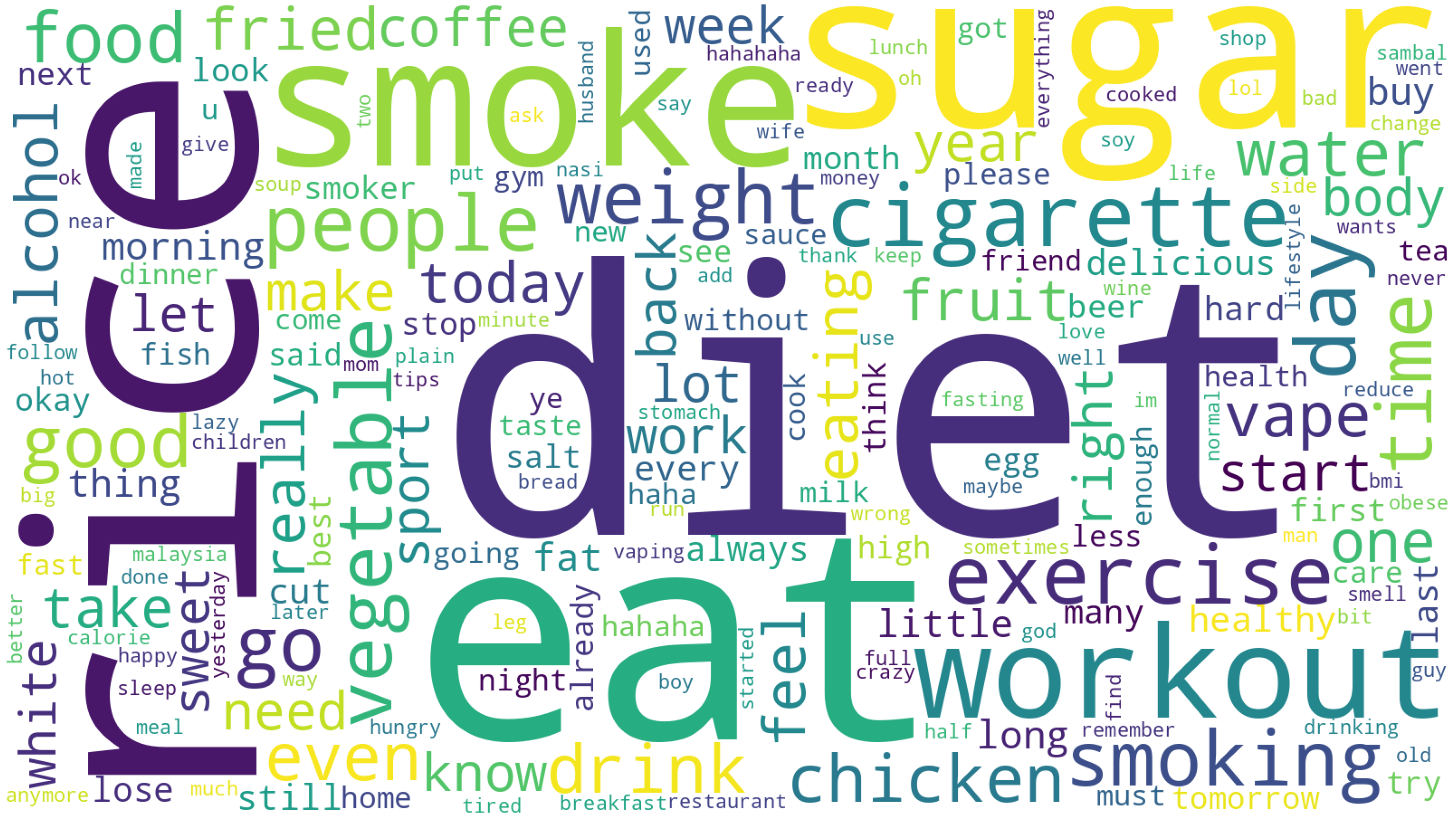

Supplement: Multimedia Appendix 3 [file infodemiology-v5-e65835-s003.png]
