# Supplementary material for: Exploring Social Media Posts on Lifestyle Behaviors: Sentiment and Content Analysis
Source: JMIR Infodemiology. 2025 Jun 25;5:e65835. doi: 10.2196/65835 (PMC12221188; doi:10.2196/65835)
Supplement: Multimedia Appendix 4 [file infodemiology-v5-e65835-s004.docx]

**Multimedia Appendix 3.** Examples of posts with positive and negative sentiments according to each lifestyle behavior.

| **Tobacco-related posts** | | | | |
| --- | --- | --- | --- | --- |
| **Post ID** | **Original post** | **Translated post** | **Score** | **Sentiment class** |
| T-510 | Jangan cari lelaki rare tak smoke/vape. Carilah lelaki yang sentiasa di rumah, kemas rumah, jaga anak, memasak, tidak keluar bekerja kuat tapi kaya raya. Nescaya anda dpt mengecapi Eutopian Happiness yang anda idamkan | Don't look for a rare man who doesn't smoke/vape. Look for a man who is always at home, cleans the house, takes care of the children, cooks, doesn't go out to work hard but is rich. You will surely be able to taste the Eutopian Happiness you desire. | 0.9643 | positive |
| T-127 | Oncall ICU ni, bila dapat baring kejap tapi malas nak tidur walaupun letih & mengantuk gila babi, sebab confirm beberapa minit lepas lelap mata mesti telefon bunyi balik. Tidur sangkut2 ni menyakitkan hati ja. P/s: musibat mana yg vape dlm bilik oncall ni?! | This ICU oncall, if you can lie down for a while but are too lazy to sleep even though you are tired and sleepy, because you confirm a few minutes after closing your eyes, the phone must ring back. Sleeping late hurts my heart. P/s: who is the one who has been vaping in this oncall room?! | -0.9493 | negative |
| **Alcohol-related posts** | | | | |
| **Post ID** | **Original post** | **Translated post** | **Score** | **Sentiment class** |
| A-41 | Happy holidays. Hope you have a great time however you celebrate. Just finished a Filipino style Noche Buena midnight feast here. Full of food and wine 🍗🍚🍷🎄🎅 https://t.co/hrGpo0l8I5 | Happy holidays. Hope you have a great time however you celebrate. Just finished a Filipino style Noche Buena midnight feast here. Full of food and wine 🍗🍚🍷🎄🎅 https://t.co/hrGpo0l8I5 | 0.9517 | positive |
| A-198 | Apologies for an evening of shit posting. I am going to drown my sorrows in alcohol and pick things back up tomorrow https://t.co/vQX1nPqAdQ | Apologies for an evening of shit posting. I am going to drown my sorrows in alcohol and pick things back up tomorrow https://t.co/vQX1nPqAdQ | -0.9720 | negative |
| **Dietary-related posts** | | | | |
| **Post ID** | **Original post** | **Translated post** | **Score** | **Sentiment class** |
| D-1493 | Mood I selalu helokkk and happy2 je sejak consume. Memang betullah supplement ni boleh menenangkan fikiran. Dah la sedapppp rasa buah markisa, nak makan pun seronok haha #EskayvieMindtropic #JanganStresStres | My mood is always happy and happy since consuming this supplement. It is true that this supplement can calm the mind. The taste of passion fruit is delicious, it's fun to eat haha #EskayvieMindtropic #JanganStressStress | 0.9764 | positive |
| D-1334 | Bawak bekal salah, diet salah, makan sikit salah, bungkus salah, makan seorang salah. Apa lagi yang korang nak? Semua benda pun salah. Geram je aku | Bring the wrong food, eat the wrong diet, eat the wrong food, pack the wrong food, eat the wrong person. What else do you want? Everything is wrong. I'm angry | -0.9666 | negative |

**Multimedia Appendix 3.** Examples of posts with positive and negative sentiments according to each lifestyle behavior. (continued)

| **Activity-related posts** | | | | |
| --- | --- | --- | --- | --- |
| **Post ID** | **Original post** | **Translated post** | **Score** | **Sentiment class** |
| P-49 | 2023 nak kurus, cantik, flawless, side income byk, duit kepuk-kepuk, savings 12k, gaji naik, technical skills mantap, workout everyday, baju size XS, grad masters. Itu je please semoga dipermudahkan, aamiin 🥹 | 2023 I want to be thin, beautiful, flawless, big side income, big money, 12k savings, salary increase, strong technical skills, workout everyday, size XS shirt, grad masters. That's it please, hopefully it will be made easier, amen 🥹 | 0.9633 | positive |
| P-402 | Jalan susah, kaki lutut sakit, semput teruk, badan sakit-sakit, nak naik tangga seksa, nak jalan jauh pun seksa. Takde rasa nak turunkan berat badan ke? Clearly your weight is making you suffered. Kesianlah orang macam tu. | The road is difficult, legs and knees hurt, wheezing is bad, body aches, it's a pain to climb the stairs, it's a pain to walk a long way. Don't feel like losing weight? Clearly your weight is making you suffer. Pity people like that. | -0.9684 | negative |

Post ID for tobacco-related posts denoted by T, alcohol-related posts denoted by A, dietary-related posts denoted by D, activity-related posts denoted by A.
