# Supplementary material for: Exploring Social Media Posts on Lifestyle Behaviors: Sentiment and Content Analysis
Source: JMIR Infodemiology. 2025 Jun 25;5:e65835. doi: 10.2196/65835 (PMC12221188; doi:10.2196/65835)
Supplement: Multimedia Appendix 5 [file infodemiology-v5-e65835-s005.docx]

**Multimedia Appendix 4.** Example of posts selected for manual content analysis.

| **Post content:** Self-narrative of current lifestyle behaviors | | | | |
| --- | --- | --- | --- | --- |
| **Sentiment** | **Post ID** | **Original post** | **Translated post** | **Alignment with recommended health practices** |
| Positive | T-271 | Don’t worry I hate perokok and will never merokok sebab semput 😍👍 | Don't worry I hate smokers and will never smoke because of wheezing 😍👍 | Aligned |
| Negative | A-203 | Ok I'm so anxious. My family slept off and I'm in the hall, with some vodka, crying over the results. COME OVER AND CRY WITH ME. #GE15 | Ok I'm so anxious. My family slept off and I'm in the hall, with some vodka, crying over the results. COME OVER AND CRY WITH ME. #GE15 | Not aligned |
| Positive | D-1267 | I love the fact that all of my Instagram friends support on my diet and eating healthy phase. | I love the fact that all of my Instagram friends support my diet and eating healthy phase. | Aligned |
| Negative | P-301 | Duduk rumah makin drop pula berat badan aku 💀 | Sitting at home makes my weight drop even more 💀 | Perceptions not defined |
| **Post content:** Narrative of others’ current lifestyle | | | | |
| **Sentiment** | **Post ID** | **Original post** | **Translated post** | **Alignment with recommended health practices** |
| Negative | T-15 | Kimak kawan finish all my rokok perghhhh 😭😭 | My friends finished all my cigarettes 😭😭 | Not aligned |
| Positive | A-254 | Makcik pakcik cheers minum apple juice dalam wine glass ehk. Comel. | Aunt uncle cheers drinking apple juice in a wine glass ehk. Cute. | Aligned |
| Negative | D-1218 | Bengang betul Fatin Sophina sampai minta kedai ni tambah garam kat telur dadar dia | Fatin Sophina was really upset till she asked this shop to add salt to her omelette | Not aligned |
| Positive | P-345 | Aku kagum dengan lawyer yg ada masa untuk ke gym dan bersukan. | I'm impressed with lawyers who have time to go to the gym and do sports. | Aligned |
| **Post content:** Planned action related to lifestyle behaviors | | | | |
| **Sentiment** | **Post ID** | **Original post** | **Translated post** | **Alignment with recommended health practices** |
| Positive | T-57 | About to get my first vape 🤭 | About to get my first vape 🤭 | Not aligned |
| Negative | A-26 | It's one of those days that desperately calls for stopping gin. | It's one of those days that desperately calls for stopping gin. | Aligned |
| Positive | D-1114 | Tonight eat a bit lesser.. keeping stomach for junk food hahahaha | Tonight eat a bit lesser.. keeping stomach for junk food hahahaha | Not aligned |
| Negative | P-522 | Terasa berat badan ..naik mendadak. Sedih. Kena perbaiki balik | Feel the weight ..rise suddenly. Sad. Have to fix it | Aligned |

**Multimedia Appendix 4.** Example of posts selected for manual content analysis. (continued)

| **Post content:** Recommendations related to lifestyle behaviors | | | | |
| --- | --- | --- | --- | --- |
| **Sentiment** | **Post ID** | **Original post** | **Translated post** | **Alignment with recommended health practices** |
| Negative | T-70 | @anthonyloke kena pantau system bas ekspres. Asyik delayed je, pastu boleh pulak berhenti tepi untuk orang turun hisap rokok. Siap boleh beli bungkus air lagi. Apa ke bodoh driver bas ekspres camni | @anthonyloke has to monitor the express bus system. Always delayed and can stop for people to get off to smoke a cigarette. Even allow passengers to buy water. What a stupid express bus driver. | Not aligned |
| Positive | A-17 | @Rich_Cooper @zahin__ So don’t do alcohol and drugs. 💯 | @Rich_Cooper @zahin__ So don't do alcohol and drugs. 💯 | Aligned |
| Negative | D-395 | Kacang, kelapa /air kelapa, buah potong. Kalau takde niat nak beli, jangan sentuh pun!!! | Nuts, coconut / coconut water, cut fruit. If you don't intend to buy, don't even touch it!!! | Perceptions not defined |
| Positive | P-349 | It’s supposed to be 12-3-30 workout. Nevermind, let’s focus on some weight lifting. | It's supposed to be 12-3-30 workout. Never mind, let's focus on some weight lifting. | Aligned |
| **Post content:** Direct question | | | | |
| **Sentiment** | **Post ID** | **Original post** | **Translated post** | **Alignment with recommended health practices** |
| Positive | T-7 | @AmmarHamidiS aik, rokok balik ke? | @AmmarHamidiS aik, smoking again? | Perceptions not defined |
| Negative | A-212 | @washimjunior Mashallah sis apa ni arak2 arak tu kan bahaya | @washimjunior Mashallah sis, what is this alcohol? Alcohol is dangerous | Aligned |
| Positive | D-526 | Any advice on how to eat healthy AND save money? | Any advice on how to eat healthy AND save money? | Aligned |
| Negative | P-678 | Kenapa selalu tak ada mood nak exercise? 😩 | Why is there always no mood to exercise? 😩 | Not aligned |
| **Post content:** General statement | | | | |
| **Sentiment** | **Post ID** | **Original post** | **Translated post** | **Alignment with recommended health practices** |
| Negative | T-699 | Tak merokok tapi hisap vape. Bahaya | Doesn’t smoke but vape. Dangerous | Aligned |
| Positive | A-263 | Free beer is always better! | Free beer is always better! | Not aligned |
| Negative | D-508 | So afraid of sugar | So afraid of sugar | Perceptions not defined |
| Positive | P-276 | Exercise harini 🤪 | Exercise today 🤪 | Aligned |

Post ID for tobacco-related posts denoted by T, alcohol-related posts denoted by A, dietary-related posts denoted by D, activity-related posts denoted by A.
